# Validated LC-MS/MS assay for quantification of the newly approved tyrosine kinase inhibitor, dacomitinib, and application to investigating its metabolic stability

**DOI:** 10.1371/journal.pone.0214598

**Published:** 2019-04-04

**Authors:** Ali S. Abdelhameed, Adnan A. Kadi, Mohamed W. Attwa, Haitham AlRabiah

**Affiliations:** 1 Department of Pharmaceutical Chemistry, College of Pharmacy, King Saud University, Riyadh, Kingdom of Saudi Arabia; 2 Students’ University Hospital, Mansoura University, Mansoura, Egypt; University of South Alabama Mitchell Cancer Institute, UNITED STATES

## Abstract

Dacomitinib (DMB) is a second-generation irreversible tyrosine kinase inhibitor (TKI) that is claimed to overcome the disadvantages of the resistance reported for first-line epidermal growth factor receptor (EGFR) TKIs. Towards the end of 2018, the US Food and Drug Administration approved DMB in the form of VIZIMPRO tablets. In the current study, a validated LC-MS/MS assay was established for DMB quantification in rat liver microsomes (RLMs) with application to the drug metabolic stability assessment. Chromatographic resolution of DMB and lapatinib (internal standard) was achieved using an isocratic mobile phase and a reversed-phase C_18_ column. The linearity of the established LC-MS/MS assay ranged from 2 to 500 ng/mL with *r*^*2*^ ≥ 0.9999. The limit of detection (*LOD*) and limit of quantification (*LOQ*) were 0.35 and 1.1 ng/mL, respectively. The precision and accuracy (both intra-day and inter-day) were 0.84–3.58% and 92.2–100.32%, respectively. The metabolic stability of DMB in the RLM matrix was estimated by calculating two parameters, *in vitro* t_1/2_ (0.97 mL/min/kg) and intrinsic clearance (157.5 min). Such values infer that DMB would be excreted very slowly from the human body, which might lead to possible bioaccumulation. To the best of our knowledge, this is the first method for DMB analysis in RLMs with metabolic stability estimation.

## Introduction

Lung cancer is the leading cause of death among all cancer types, in particular, non-small cell lung cancer (NSCLC) is considered the most widespread [1–5], with an incidence of approximately 90%. The epidermal growth factor receptor (EGFR) signaling pathway has gained importance in the last few years as a therapeutic target for NSCLC [6]. Tyrosine kinase inhibitors (TKIs) that control EGFR are very efficient in the treatment of cancers possessing EGFR mutations, with a characteristic therapeutic window. First-line TKIs controlling EGFR (e.g., erlotinib and gefitinib) have good initial responses against these mutations [7, 8]. Unfortunately, acquired resistance in ~60% of patients and toxicities that occur during treatment [9, 10] decrease their therapeutic efficacies [11, 12]. This has led scientists to develop second-generation, irreversible EGFR TKIs (e.g., dacomitinib (DMB) and avitinib) [13, 14].

**Fig 1:**
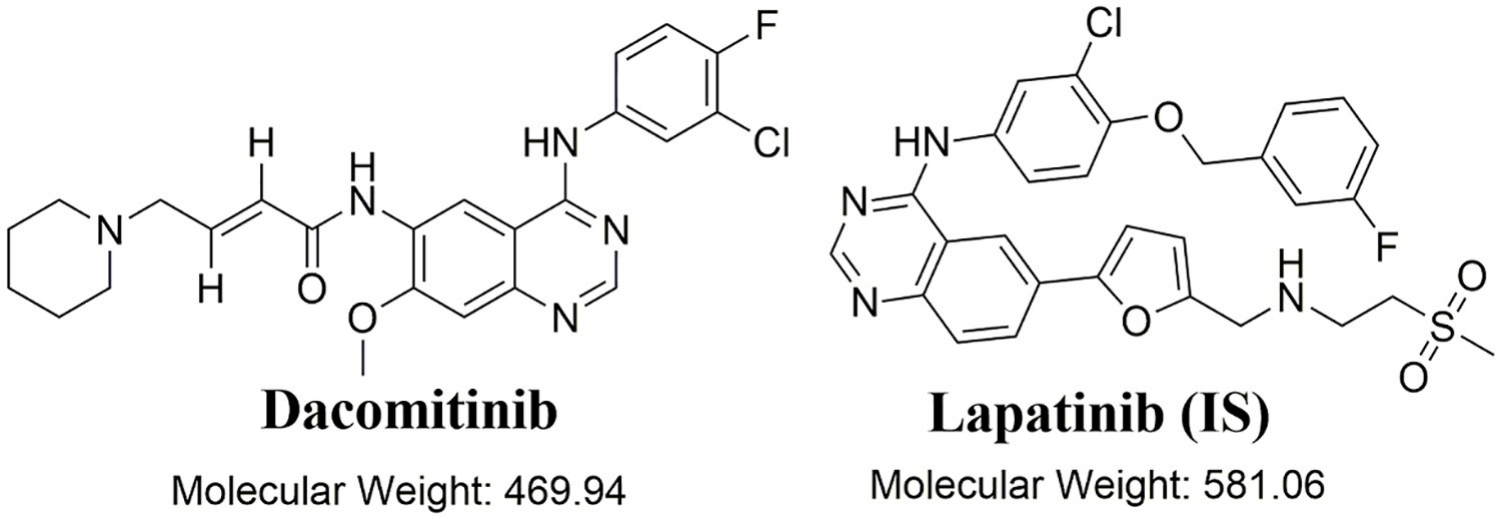
Chemical structures of dacomitinib and lapatinib (IS).

DMB (Fig 1) overcomes the acquired resistance observed with first-line EGFR TKIs [13–15]. It was shown to improve progression-free survival when compared with that of gefitinib in the treatment of NSCLC patients with positive EGFR mutations. This represents a new achievement for the treatment of these patients [16]. On September 27, 2018, the Food and Drug Administration (FDA) approved DMB in the form of VIZIMPRO tablets for the first-line treatment of patients with metastatic NSCLC harboring EGFR exon 19 deletions or exon 21 L858R substitution mutations [17]. In addition, a DMB marketing authorization application was accepted by the European Medicines Agency (EMA) for the same indication [18].

To the best of our knowledge, a single LC/MS-MS assay was lately published reporting the analysis of DMB in rat plasma [19]. The purpose of the present study was to establish a validated LC-MS/MS assay to quantify DMB in rat liver microsomes (RLMs) as a different biological matrix to the drug and to allow the application of this assay to investigate the DMB metabolic stability by calculating two important parameters (i.e., intrinsic clearance and *in vitro* half-life (t_1/2_)). These parameters could then be utilized for *in vivo* t_1/2_, hepatic clearance, and bioavailability calculations. Bioavailability is important because it provides information about the metabolism of the investigated compound; if the compound is rapidly metabolized, it will exhibit low bioavailability *in vivo* [20].

## Experimental

### Reagents and chemicals

All chemicals and solvents were of analytical grade. Dacomitinib (DMB) and lapatinib (internal standard; LTP; IS) were purchased from Med Chem Express (Princeton, NJ, USA). Rat liver microsomes (RLMs), Acetonitrile (ACN), ammonium formate (NH4COOH), and formic acid (HCOOH) were purchased from Sigma Aldrich (St. Louis, MO, USA). HPLC-grade water (H_2_O) was obtained from the Milli-Q plus filtration system (Millipore, Billerica, MA, USA).

### LC-MS/MS methodology

All LC-MS/MS parameters were optimized to achieve the best chromatographic resolution of DMB and IS with good separation. LTP was chosen as the IS in the DMB analysis because the same extraction procedure can be applied efficiently with a great success for both compounds (DMB and LTP recoveries were 97.91±3.74% and 97.2 ± 1.3%, respectively in the RLM matrix) and the elution time of LTP is comparable to that of DMB. The proposed procedure is rapid with 4 min run time. Both LTP and DMB are TKIs and are not co-administered to patients, so this assay might be applied for pharmacokinetics or therapeutic drug monitoring (TDM) for subjects treated with DMB.

**Fig 2:**
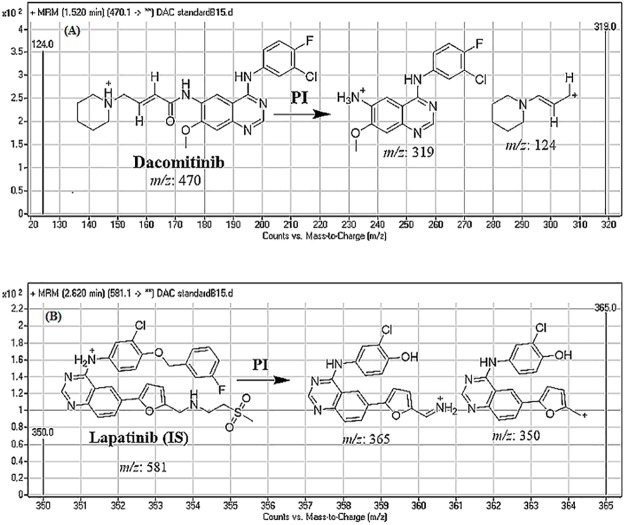
MRM mass spectrum transitions of DMB (A) and lapatinib (IS) (B).

Agilent eclipse plus C_18_ column (100 mm in length, 2.1 mm in internal diameter and 1.8 μm particle size) was used for chromatographic resolution of analytes. The column temperature was adjusted at 22±1 °C. A triple quadrupole (QqQ) mass spectrometer (Agilent Technologies, CA, USA). with an electrospray ionization source interface (ESI), running in the positive mode, was used for detection. Low purity nitrogen (11 L/min) was utilized as the drying gas in the ESI source and high purity nitrogen (55 psi) was employed as the collision gas. The values of capillary voltage (V) and ESI temperature (T) were set at 4000 V and 350°C, respectively. The instruments and data acquisition were controlled using the Mass Hunter software (Agilent Technologies, CA, USA). DMB was quantified using the multiple reaction monitoring (MRM) scanning mode, for the mass reactions (parent to fragment ions) from 470.1→319 and 470.1→124 for DMB and 581→365 and 581→365 for LTP (Scheme 1). The fragmentor voltage (FV) was set at 140 V with collision energy (CE) of 30 eV for DMB, and FV of 140 V and 145 V with CE of 30 eV and 32 eV for LTP. MRM transitions were used for determination of DMB to eliminate any interference caused by the RLM matrix components and enhance the sensitivity of the proposed LC-MS/MS analytical method (Fig 2).

### Preparation of DMB calibration standards

Both DMB and LTP are soluble in dimethyl sulfoxide (DMSO). DMB was dissolved in DMSO at 2 mg/mL. Afterwards, this stock solution was diluted 10 times with mobile phase to prepare DMB solution 1 (200 μg/mL), which was then diluted 10 times with mobile phase to prepare DMB solution 2 (20 μg/mL). LTP stock solution (100 μg/mL) was prepared in DMSO and then diluted fifty times with the mobile phase to prepare LTP (IS) solution 3 (2 μg/mL). DMB solution 2 (20 μg/mL) was diluted with specific RLM matrix (40 μL in 1 mL of phosphate buffer at pH: 7.4) to generate fifteen calibration standards: 2, 5, 10, 15, 20, 30, 40, 50, 80, 100, 150, 200, 300, 400, and 500 ng/mL. Four standards (2, 15, 150, and 400 ng/mL) were selected as quality controls, namely the lower limit quality control (LLQC), low quality control (LQC), medium quality control (MQC), and high quality control (HQC), respectively.

### Extraction of DMB from RLMs matrix

Analytes extractions were performed using ACN protein precipitation, a standard technique for metabolic stability experiments [21]. Specifically, 2 mL ACN was added to each mL of calibration standard followed by centrifugation at 14000 rpm (12 min at 4 °C) to deproteinize by precipitation [22]. All supernatants were then filtered using syringe filters (0.22 μm pore size). Fifty microliters of IS solution 3 was added to 1 mL of each filtered samples and transferred to 1.5 mL vials. Two microliters of each sample was then injected into the LC-MS/MS for analysis. Similarly, blank samples were prepared by using the stated phosphate buffer without RLM matrix to confirm that RLM components did not interfere with the elution time of DMB and IS. A calibration curve was established by plotting the peak area ratio of DMB to IS (*y* axis) against the nominal values (*x* axis). A linear regression equation was used to validate the linearity of the established assay. Slope, intercept, and coefficient of determination (*r*^*2*^) values were computed.

### Method validation

The established LC-MS/MS assay was validated for sensitivity, assay recovery, linearity, reproducibility, specificity, limit of quantification (LOQ), limit of detection (LOD). and stability according to the US Food and Drug Administration (FDA) guidelines [23]. The validation parameters of the LC-MS/MS assay that was developed to quantify DMB were described in more detail previously [22, 24]. These parameters included the least squares statistical method was utilized to calculate the calibration curve equations (*y = ax + b*). The linear fit was verified using the *r*^*2*^ value.

### Metabolic stability of DMB

The metabolic stability study for DMB was performed by assessing the decrease in DMB concentration after incubation with RLMs. One micromolar DMB was incubated with RLMs (1 mg microsomal protein / 1 mL of phosphate buffer) in triplicate. The medium for metabolic reaction was phosphate buffer (pH 7.4) containing 3.3 mM MgCl_2_. The mixture was pre-incubated for 10 min in a 37 °C water bath. The metabolic reaction was initiated by adding NADPH (1 mM) and was terminated by adding 2 mL ACN at specific time intervals (0, 2.5, 5, 7.5, 10, 15, 20, 40, and 50 min). ACN was used for stopping the metabolic reaction and protein precipitation method that was used for extraction of DMB from RLMs incubation. All incubates were centrifuged at 14000 rpm (12 min at 4 °C). All supernatants were then filtered using syringe filters (0.22 μm pore size). Fifty μL of IS solution 3 was added to 1 mL of each filtered samples and transferred to 1.5 mL vials. Two microliters of each sample was then injected into the LC-MS/MS for analysis. The metabolic stability curve for DMB was then created.

## Results and discussion

### HPLC–MS/MS methodology

Optimization of chromatographic parameters, including the mobile phase pH, mobile phase constituents, and C_18_ column, was performed. The pH of the aqueous portion (10 mM NH_4_COOH) was adjusted to 4.2 with formic acid. A pH above this value caused peak tailing and an unnecessary increase in retention time. The ratio of the aqueous portion to the organic portion (ACN) of the mobile phase was adjusted to 30%: 70%; higher volume of ACN resulted in overlapping of the chromatographic peaks with bad peak resolution while reducing the ACN volume <70% resulted in longer run times. RRLC columns with different types of stationary phases (e.g., HILIC columns) were tested, but both DMB and IS were not retained on such columns; the unsurpassed results were obtained using C_18_ column. DMB was quantified using MRM from 470.1→319 and 470.1→124 for DMB and 581→365 and 581→365 for IS. The MRM detection mode of the mass spectrometer was utilized for the quantification of DMB ions to avoid potential interference from the RLM matrix constituents and improve the sensitivity of the established LC-MS/MS assay (Fig 2).

**Fig 3:**
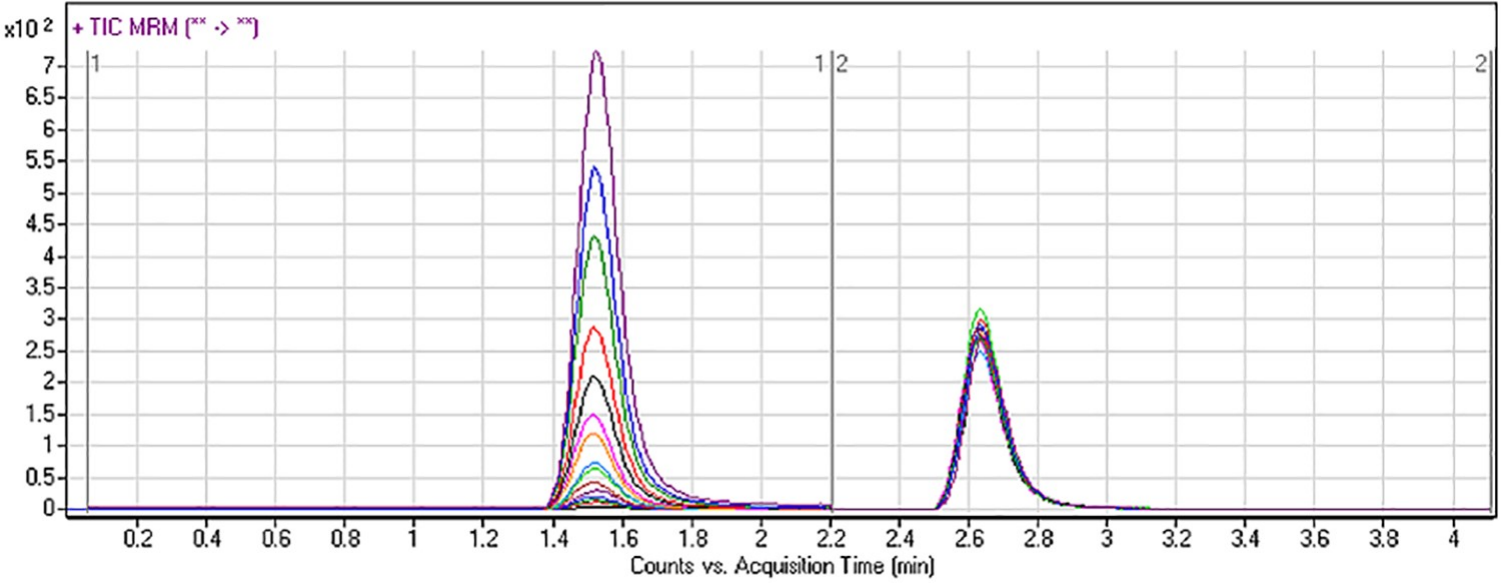
Overlaid MRM chromatograms of fifteen calibration standards of DMB (2–500 ng/mL) and IS (100 ng/mL).

The chromatographic separation of DMB and IS was achieved in 4 min. DMB and IS chromatographic peaks eluted at 1.5 min. and 2.6 min, respectively. The analytes peaks were well resolved, with no carryover to the blank samples (i.e., RLM matrix samples or RLM plus IS samples). Fig 3 shows the overlaid MRM chromatograms of 15 DMB calibration standards, showing concentration-dependency and consistency in their profiles.

### Validation of the developed LC-MS/MS assay

#### Specificity

**Fig 4:**
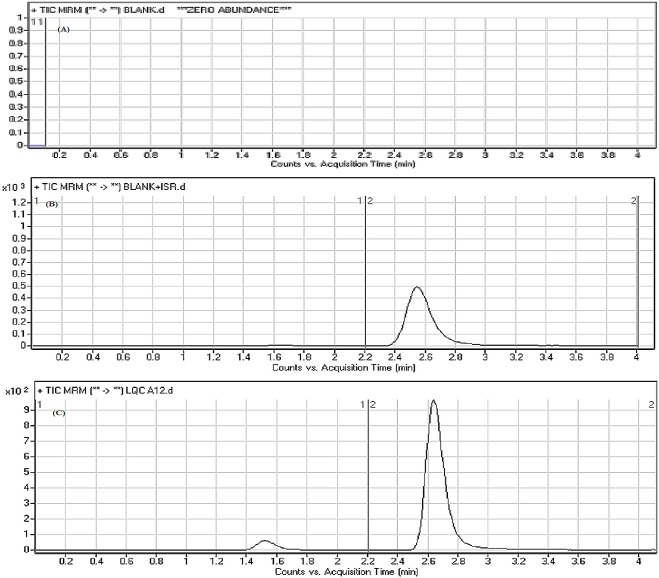
MRM chromatograms of blank (A), blank + IS (B), and DMB 15 ng/mL LQC (C). The blank RLM matrix revealed no matrix interference.

Fig 4 reveals good separation of the DMB and IS peaks and the absence of peaks with the blank RLM matrix at the corresponding retention times, which supports the specificity of the developed methodology. No carryover effect of DMB and IS was observed in the MS chromatograms.

#### Sensitivity and linearity

**Fig 5:**
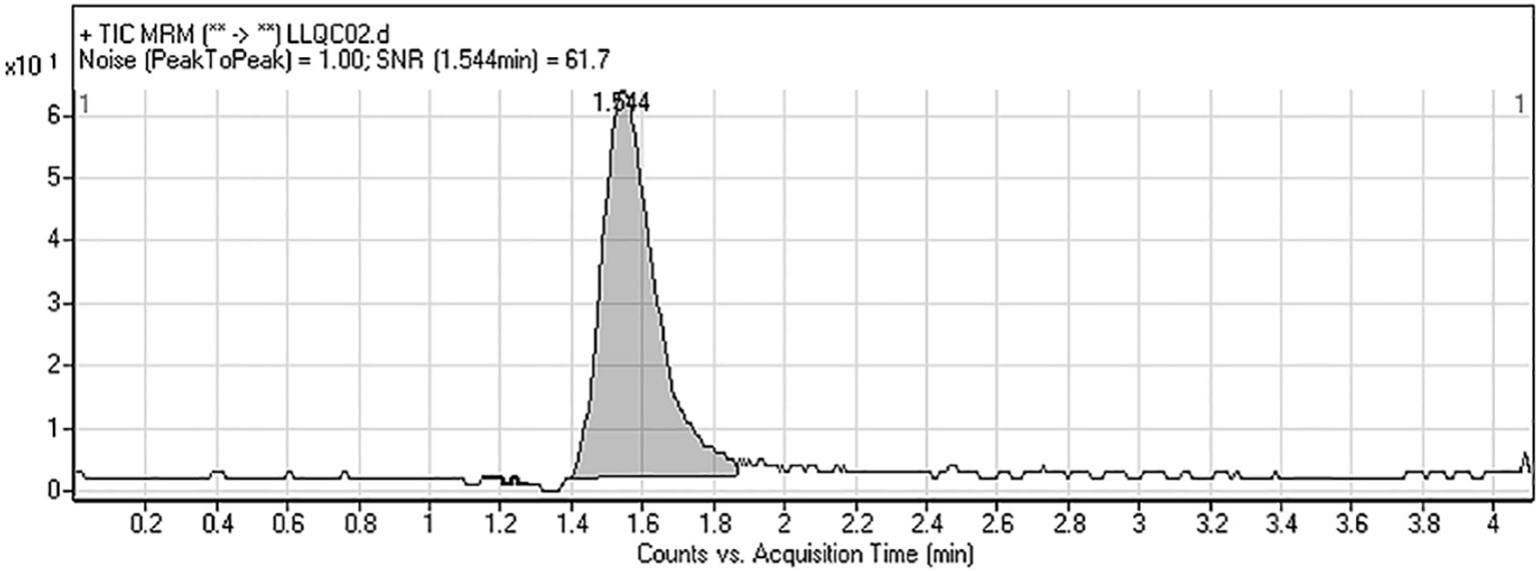
DMB LLQC MRM chromatogram revealing high *S/N*.

The linear range and correlation coefficient (*r*^*2*^) for the proposed methodology were 2–500 ng/mL and ≥ 0.9999 in the RLM matrix, respectively. The regression equation of the DMB calibration curve was *y* = 0.4919*x* + 0.6092. The *LOD* and *LOQ* were equal to 0.35 and 1.1 ng/mL, respectively. The LLQC peak showed a very high signal-to-noise ratio (*S/N*) and good peak shape that validated the sensitivity of the developed LC-MS/MS assay (Fig 5).

#### Extraction recovery

The RSD values of six repetitions for each concentration level in the calibration curve were less than 1.96% in the RLM matrix (Table 1). Back calculations of the fifteen samples of DMB in the RLM matrix (Calibration standards and QC samples) confirmed the performance of the developed methodology. The recovery of DMB detected in the spiked RLM matrix samples was 97.91 ± 3.74% with a RSD of less than 1.96%.

**Table 1:** Data of back-calculated DMB concentrations for the calibration standards from the RLM matrix.

Nominal Concentrations of DMB in ng/mL	Mean^a^	SD	RSD %	Recovery %
2 (LLQC)	1.84	0.02	1.26	92.20
5	4.61	0.08	1.63	92.17
10	9.22	0.10	1.11	92.18
15 (LQC)	13.98	0.13	0.96	93.22
20	19.44	0.20	1.03	97.19
30	29.22	0.30	1.04	97.40
40	39.39	0.62	1.58	98.49
50	51.54	1.01	1.96	103.08
80	80.42	1.12	1.39	100.52
100	101.55	0.44	0.44	101.55
150 (MQC)	150.47	1.32	0.88	100.32
200	199.81	1.78	0.89	99.90
300	303.26	1.10	0.36	101.09
400 (HQC)	395.90	3.34	0.84	98.98
500	502.12	2.97	0.59	100.42
**Average** ± SD				97.91±3.74

^a^ Average of six replicates

#### Precision and accuracy

Considering both intra-day and inter-day values, the precision and accuracy ranged from 0.84 to 3.58% and 92.20 to 100.32%, respectively (Table 2). The mean percentage of DMB recovery was 97.91 ± 3.74% in the RLM matrix (Table 1). The intra- and inter-day accuracy and precision values are acceptable according to International Conference for Harmonization (ICH) guidelines [25, 26].

**Table 2:** Precision and accuracy (intra-day and inter-day) of the developed assays.

RLM matrix	LLQC (2 ng/mL)		LQC (15 ng/mL)		MQC (150 ng/mL)		HQC (400 ng/mL)	
	Intra-day assay*	Inter-day assay**	Intra-day assay	Inter-day assay	Intra-day assay	Inter-day assay	Intra-day assay	Inter-day assay
**Mean**	1.84	1.86	13.98	13.95	150.47	148.96	395.90	394.33
**SD**	0.02	0.07	0.13	0.26	1.32	2.51	3.34	4.26
**Precision (%RSD)**	1.26	3.58	0.96	1.84	0.88	1.69	0.84	1.08
**% Accuracy**	92.20	92.93	93.22	93.02	100.32	99.31	98.98	98.58

* Average of twelve replicates from day 1.

** Average of six replicates from three consecutive days

#### Matrix effects

The absence of an RLM matrix effect was confirmed by analyzing six different batches of RLM matrixes form six different rats, in which these batches were extracted and spiked with DMB (LLOQ (15 ng/mL), LQC (15 ng/mL), MQC (150 ng/mL) and HQC (400 ng/mL)) and IS. The abovementioned batches were named set 1. Set 2 was prepared in a similar way, except utilizing the mobile phase instead of the RLM matrix. Thus, the matrix effect was calculated from the following equation:

Matrixeffect=MeanpeakarearatioSet1Set2×100

The studied RLM matrix that contained DMB had a matrix effect of 94.46 ± 2.94%. Accordingly, these results reveal that the influence of the RLM matrix on DMB and IS (IS) ionization was low (Table 3).

**Table 3:** RLMs matrix effect on the DMB analysis.

Nominal Conc. ng/mL	2 ng/mL	15 ng/mL	150 ng/mL	400 ng/mL	Average ± SD
**Mean**^a^	1.80	14.22	144.21	386.87	
**SD**	0.04	0.23	1.71	4.95	
**Precision (RSD %)**	1.98	1.64	1.18	1.28	
**Recovery (%)**	90.22	94.77	96.14	96.72	94.46 ± 2.94

^**a**^ Average of six replicates

### Stability

The stability of DMB in RLMs matrix was evaluated under all conditions that might have been encountered before the analysis. DMB displayed good stability in RLMs matrix samples after storage at −20 °C for 28 days as stability values were ranged from 98.89 to 98.9%. Detailed stability data for DMB is summarized in Table 4. There was no observed degradation of analytes under the tested conditions indicating that DMB displayed good stability in all analyses.

**Table 4:** Stability of dacomitinib (DMB) under different storage conditions.

Nominal Concentrations of DMB in ng/mL	Mean^a^	SD	RSD %	Accuracy %
**Room Temp. for 8 hr**				
2	1.87	0.06	3.28	93.59
15	14.72	0.34	2.34	97.30
150	147.46	1.35	0.92	98.31
400	393.12	3.52	0.90	97.72
**Three freeze-thaw cycles**				
2	1.82	0.04	2.45	91.10
15	14.29	0.09	0.62	95.29
150	144.21	4.88	3.39	96.14
400	385.87	2.68	0.69	96.47
**Sored at 4 °C for 24 hr**				
2	1.83	0.03	1.57	91.47
15	14.22	0.23	1.64	94.77
150	145.71	2.46	1.69	97.14
400	387.12	4.08	1.05	96.78
**Sored at -20 °C for 30 days**				
2	1.80	0.06	3.56	89.98
15	14.39	0.57	3.98	95.95
150	148.21	1.85	1.25	98.81
400	392.37	4.14	1.05	98.09

^a^ Average of six replicates

### Metabolic stability

**Fig 6:**
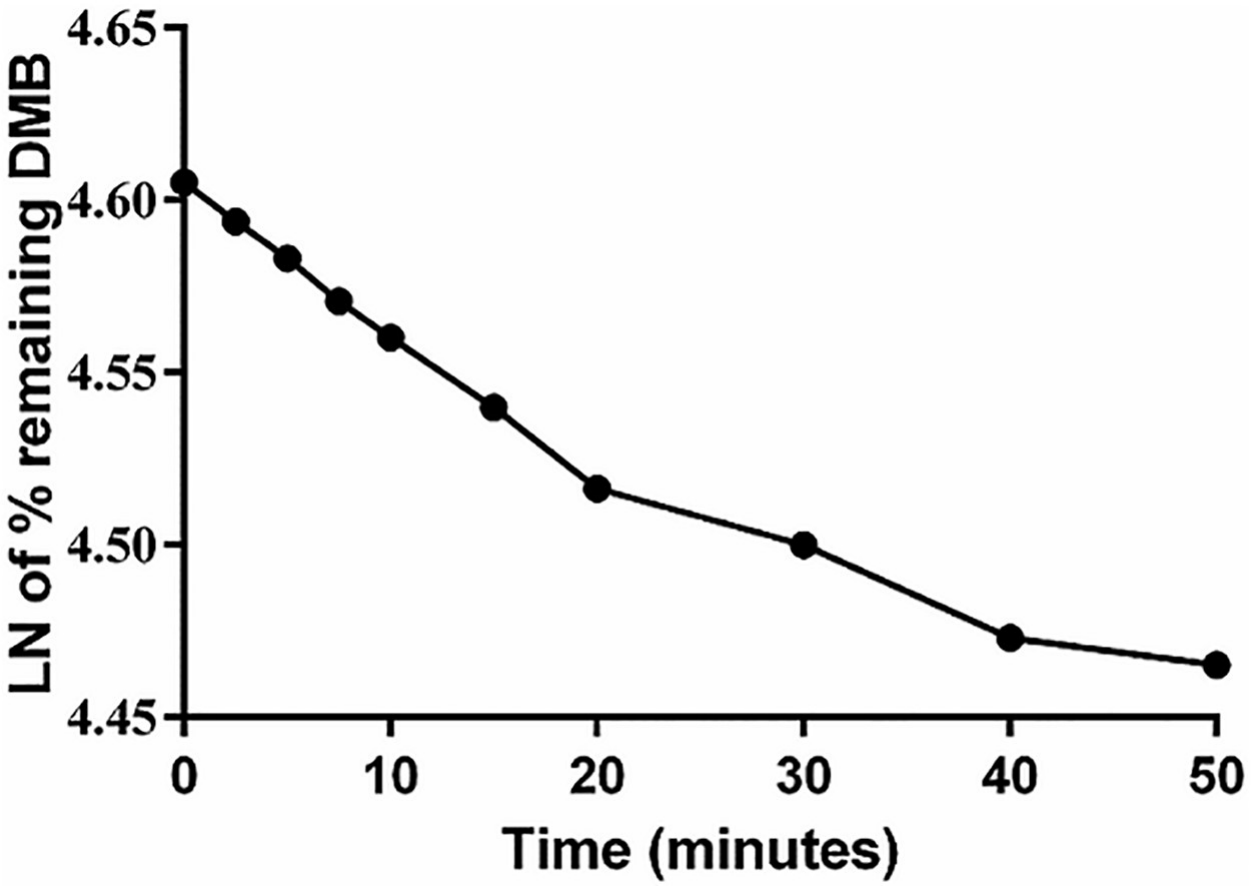
Metabolic stability curve of DMB treated with RLMs.

The DMB concentration in the RLM matrix was calculated by the displacement of the peak area ratios in the regression equation of the calibration curve. A metabolic stability curve was drawn by plotting the natural log (Ln) of the remaining percent of DMB on the *y*-axis against incubation time on the *x*-axis (Fig 6). The linear portion of the plotted curve was used to calculate *in vitro t*_*1/2*_ [27]. The regression equation for this linear region was *y* = -0.0044*x* + 4.6048 with *r*^*2*^ = 0.9989 (Table 5).

**Table 5:** Metabolic stability parameters for DMB incubation with RLMs.

OTB metabolic stability parameters	
Parameter	Value
Regression equation ^a^	*y* = -0.0044*x* + 4.6048
*r*^*2*^ ^b^	0.9989
Slope	0.0044
*t*_*1/2*_ ^c^	157.5 min
*CL*_*int*_ ^d^	0.97 mL/min/kg

^a^ Regression equation of linear portion of curve

^b^ Correlation coefficient

^c^ Half-life

^d^ Intrinsic clearance.

By using the following equation, where the slope was 0.0044:

Invitrot1/2=ln2Slope

Invitrot1/2=ln20.0044

$${In\;vitro\;t\;}_{1/2}=157.5\;min.$$

The intrinsic clearance (CL_int_) of DMB was calculated, according to the *in vitro t*_*1/2*_ method [20], using the following equation:

$${CL}_{int,app}=\;\frac{0.693}{{in\;vitro\;t\;}_{1/2}}.\frac{\text{mL}\;\text{incubation}}{\text{mg}\;\text{microsomes}}\;.\frac{45\;\text{mg}\;\text{microsome}}{\text{g}\;\text{liver}}\;.\frac{20\;\text{g}\;\text{liver}}{\text{kg}\;\text{per}\;\text{body}\;\text{weight}}$$

$${CL}_{int,app}=\frac{0.693}{157.5}\;.\;\frac{1}{1}\;.\frac{45}{12.5}\;.\frac{20\;}{0.325}$$

$${CL}_{int,app}=0.97\;\text{mL}/\text{min}/\text{kg}$$

From these results, the metabolic stability of DMB was characterized by a very low *CL*_*int*_ (0.97 mL/min/kg) and a very long *in vitro t*_*1/2*_ (157.5 min), which resulted in a very slow clearance of DMB from the blood by the liver. This probably resulted in a very high *in vivo* bioavailability that corroborated the high oral bioavailability previously reported and also indicated that DMB would possibly be bioaccumulated after multiple doses [28].

## Conclusions

A validated LC-MS/MS methodology was developed for quantifying the newly approved drug, DMB. The established assay is highly sensitive (*LOD* = 0.35 ng/mL), eco-friendly (small volume of ACN), fast (run time = 4 min.), accurate (92.2 to 100.32%), and has high recovery (97.91±3.74%). The LC-MS/MS assay was applied for DMB metabolic stability assessment with an RLM matrix, and the two parameters, *in vitro t*_*1/2*_ (157.5 min) and *CL*_*int*_ (0.97 mL/min/kg), were calculated. Accordingly, with such high *t*_*1/2*_ and low *CL*_*int*_ values, DMB can be further investigated for its drug plasma concentration and effect on kidney function due to possible drug bioaccumulation as it might have a very low extraction ratio with a very slow excretion.
